# Personalized Medicine in Brain Tumors

**DOI:** 10.3390/jpm14040413

**Published:** 2024-04-13

**Authors:** Quintino Giorgio D’Alessandris, Martina Offi, Liverana Lauretti, Roberto Pallini

**Affiliations:** 1 Department of Neuroscience, Catholic University School of Medicine, 222 Banpo-daero Seocho-gu, Seoul 06591, Republic of Korealiverana.lauretti@unicatt.it (L.L.); roberto.pallini@unicatt.it (R.P.); 2 Department of Neurosurgery, Fondazione Policlinico Universitario A. Gemelli IRCCS, Largo A. Gemelli, 8, 00168 Rome, Italy

Personalizing clinical, diagnostic and therapeutic approaches in neuro-oncology is a huge challenge. Brain cancer has been the focus of intense research since the beginning of the third millennium, with a particular interest in glioblastoma, the most frequent and malignant brain tumor in adults. Glioblastoma was the first tumor to be studied in the NIH-funded The Cancer Genome Atlas Program [1], unraveling its unthinkable molecular heterogeneity. In the meantime, cellular heterogeneity was addressed through the identification of a population of glioblastoma stem-like cells (GSCs) [2] and through the definition of the different transcriptional programs active in separate subpopulations of glioblastoma cells [3]. In the context of such a steadily growing body of knowledge on brain tumors, about a decade ago, molecular classifications started to emerge, albeit with no therapeutic and uncertain prognostic implications [4]. Indeed, adult neuro-oncology was experiencing particularly harsh times, encompassing the failure of multiple trials with molecularly targeted drugs [5,6]. Mild signs of hope came from the revised fourth edition of the World Health Organization Classification of Tumours of the Central Nervous System [7], in which, for the first time, molecular information was combined with histopathological findings to generate an “integrated” diagnosis with therapeutic implications. This attitude was reinforced in the novel fifth edition of the classification [8], in which the key role of the driver mutations of some families of brain tumors (e.g., isocitrate dehydrogenase, IDH, for lower-grade gliomas, or mitogen-activated protein kinase, MAPK, in certain pediatric low-grade gliomas) was pinpointed. From a therapeutic viewpoint, after the failure of countless trials, new enthusiasm was raised as a result of the REGOMA trial, in which, for the first time, a targeted drug, the multikinase inhibitor regorafenib, showed some effectiveness in treating recurrent glioblastoma [9]. While there is a long road ahead, the current feeling is that, finally, we are on the right path to find an effective treatment for brain tumors. It is essential to keep a multidisciplinary and multimodal approach, by combining different research and omic modalities, by coordinating the work of basic science researchers and physicians coming from different medical specialties, and by integrating several tailored therapeutic strategies. Next-generation sequencing tools, animal facilities, operating rooms, linear accelerators, chemotherapeutic drugs, and immunotherapy approaches must all be combined in a tailored fashion for each tumor and each patient to achieve success in neuro-oncology.

In the Special Issue Personalized Medicine in Brain Tumors of the Journal of Personalized Medicine, we aimed at giving up-to-date, state-of-the-art personalized medicine efforts in neuro-oncology, with the purpose to foster novel studies in this field. The papers were focused mainly on glioblastoma, an incurable tumor with a grim prognosis [10]. However, other tumors as well as multidisciplinary care and end-of-life treatments were addressed in this issue.

In Contribution 1, Gilard et al. provided an overview of glioblastoma care. The current management of glioblastoma envisages maximal safe surgical resection followed by adjuvant radiotherapy and chemotherapy. Despite notable advances in our understanding of glioblastoma tumorigenesis and progression, tumor recurrence is unavoidable, and the median overall survival does not exceed 15 months. Moreover, patients often experience a profoundly altered quality of life. The wide heterogeneity of glioblastoma implies a variable response to treatments; this highlights the great importance of identifying individualized paths of care in neuro-oncologic patients.

Preclinical research on glioblastoma was addressed in Contributions 2, 11 and 13.

The biomolecular mechanisms underlying tumor progression and resistance to therapy have been extensively studied in recent years. It is widely recognized that glioblastoma resistance to current therapies is due to a subpopulation of tumorigenic stem-like cells, the GSCs, which hierarchically drive tumor onset and progression. In Contribution 2, Vargas-Toscano et al. described a novel pipeline based on the integration of recent theoretical and experimental publications, with the aim to speed up the development of novel anti-glioblastoma treatments based on the GSC model. This approach is aimed at narrowing the gap between basic research and clinical application.

A hallmark of glioblastoma is its abundant and aberrant vasculature. Glioblastoma adopts different strategies in order to foster its vasculature, among which the most known and frequently studied is angiogenesis. Therefore, as has already happened for many tumor histotypes, in the last 20 years, anti-angiogenic treatments have been extensively explored in glioblastoma. However, many studies showed that, after some time, glioblastoma develops resistance to anti-angiogenic treatments and can become even more aggressive. In Contribution 11, Buccarelli et al. showed that an improved insight into the mechanisms of resistance to anti-angiogenic therapies (e.g., cell trans-differentiation, vascular mimicry, vessel co-option) and a better selection of patients through the discovery of predictive factors for response will be crucial to setting up effective anti-angiogenic strategies.

Histone deacetylases (HDACs) are a family of enzymes which epigenetically control gene expression at the chromatin level. HDACs eliminate acetyl groups from histone proteins, thus driving the condensation of chromatin into heterochromatin and suppressing gene transcription. Aberrant HDAC activity has been described in several diseases including cancer. Due to their key role in regulating gene expression, HDAC inhibitors hold great potential as anticancer therapies; additionally, HDAC inhibitors display a synergistic activity with DNA-damaging agents such as radiation and cisplatin, enhancing their cytotoxic effects. In Contribution 13, Drzewiecka et al. found that the HDAC inhibitor valproic acid synergized with the PARP1 inhibitor talazoparib (BMN-673), and with the alkylating agent temozolomide, to induce DNA damage and kill glioblastoma cells. This study indicates that combining HDAC inhibitors with PARP inhibitors could potentially enhance the treatment of glioblastoma.

Clinical research on glioblastoma was addressed in Contributions 3, 12, 4, 5, 7, and 9.

Gross total resection remains the gold standard for glioblastoma. In the case of unresectable tumors, few studies have investigated the clinical impact and long-term outcomes of biopsy. In Contribution 3, Di Bonaventura et al. showed a 93% concordance between radiological and histopathological diagnosis. Concerning the long-term outcome of biopsy patients, a key role of the completion of adjuvant chemoradiation was demonstrated: the median overall survival was 11 months with vs. 2 months without treatment.

Refining the current chemotherapeutic protocol for glioblastoma has been the focus of intense research. In Contribution 12, Gherasim-Morogai et al. investigated the impact of extending the duration of adjuvant temozolomide treatment beyond the six cycles prescribed by the standard of care [10]. They found that carefully selected glioblastoma patients may derive benefit from this approach.

Contributions 4, 5 and 9 from Prof. Fiorentino’s group dealt with radiotherapy. In Contribution 4, a retrospective study was conducted to evaluate the efficacy and toxicity of hypofractionated radiotherapy with a simultaneous integrated boost in association with temozolomide in glioblastoma poor prognosis patients. This treatment was shown to be safe and effective. Contribution 5 described a new immobilization solution and surface-guided radiation to facilitate the technical aspects and patient comfort during LINAC-based treatment of patients with brain tumors. In Contribution 9, the safety and efficacy of re-irradiation through radiosurgery or fractionated stereotactic radiotherapy, combined with chemotherapy, in patients with recurrent glioblastoma was evaluated. Thirty patients suffering from recurrent glioblastoma were evaluated, showing that re-irradiation in association with second-line systemic therapy is a safe and effective treatment.

In Contribution 7, Corr et al. performed a literature review on Radiogenomics, a novel field of study which establishes a link between radiological and pathological information with prognostic and therapeutic implications. MGMT status, IDH, EGFR status, molecular subgroups, and tumor location can be linked with radiological appearance, prognosis, and response to treatment in glioblastoma. By reviewing the advances in the development of radiogenomic markers, some studies tried to shed light on the potential impact of such an approach in daily clinical practice. Radiogenomics, for example, could allow a timely identification of glioblastoma recurrence, fostering personalized monitoring and treatment strategies. However, well-designed prospective radiogenomic studies are lacking.

Contribution 10 from our research group dealt with the need for adequate organizational networks for patients destined for palliative care. In a study on 25 patients, we showed that for the end of life, the majority of patients chose a hospice care facility (72%). The presence of an institutional palliative care service emerged as an essential requirement to cope with patients’ and caregivers’ needs.

Contribution 6 highlighted the role of the multidisciplinary tumor board (MTB) in providing quality cancer care, assuring the best personalized clinical approach. Gaudino et al. focused on the role of neuroradiologist, finding that imaging review by an expert neuroradiologist impacts on patient management in the context of neuro-oncologic MTBs. However, it carries non-negligible costs in terms of time and effort spent by the neuroradiology personnel for cases study.

Finally, in Contributions 8, Damodharan et al. discussed the impact of the personalized medicine approach in the so-called diffuse intrinsic pontine glioma. It is a highly malignant pediatric brain tumor which, from a histological viewpoint, displays notable cellular polymorphism and infiltrative ability. From a genetic viewpoint, histone protein mutations are the key driver mutations, with a lysine-to-methionine point mutation at position 27 (K27M) of histone 3 (H3) being the most common. Accordingly, these tumors are now defined as diffuse midline glioma, H3 K27-altered, and are graded as Grade 4 in the WHO classification [8]. Radiological diagnosis is performed using MRI and it is quite straightforward. However, in recent years, the need for biopsy sampling has steadily risen, allowing us to confirm the diagnosis and to improve our understanding of the disease mechanisms through huge international collaborative efforts. Radiation therapy remains the standard treatment option. Many chemotherapeutic agents have been tested, without significant benefit on survival over radiotherapy alone. Current efforts are focused on drugs acting at epigenetic level, on strategies to bypass the blood–brain barrier, and on immunotherapy.
